# Spore Morphology of *Platycerium* (Polypodiaceae) and Its Implications

**DOI:** 10.3390/plants15030370

**Published:** 2026-01-24

**Authors:** Dan-Ni Ma, Bin Yang, Jing Zhao, Li-Ju Jiang, Hong-Bin Niu, Shuai Yang, Jian-Rong Zhang, Xin-Mao Zhou, Zhao-Rong He, Cong-Li Xu, Jia-Guan Wang

**Affiliations:** 1 School of Life Sciences & School of Ecology and Environmental Science, Yunnan University, Kunming 650504, China; 12024230053@stu.ynu.edu.cn (D.-N.M.); zhaojing@mail.ynu.edu.cn (J.Z.); zhangjianrong@stu.ynu.edu.cn (J.-R.Z.); xinmao.zhou@ynu.edu.cn (X.-M.Z.); zhrhe@ynu.edu.cn (Z.-R.H.); 2 Yunnan Gaoligongshan National Nature Reserve, Baoshan Bureau, Baoshan 678000, China; 13769052725@163.com; 3 Centre for Gardening and Horticulture, Xishuangbanna Tropical Botanical Garden, Chinese Academy of Sciences, Menglun, Mengla 666303, China; jiangliju@xtbg.ac.cn (L.-J.J.); niuhongbin@xtbg.ac.cn (H.-B.N.); 4 Plant Fairyland, Boda Road, Kunming 650503, China; eve_yangshuai@sina.cn

**Keywords:** spore, ploidy, phylogeny, taxonomy, Platycerioideae

## Abstract

The spore characteristics of ferns play an important role in taxonomy; however, comprehensive spore data for most species of the genus *Platycerium* remain scarce. In this study, spores of *Platycerium* were examined using light microscopy and scanning electron microscopy. We established the first comprehensive dataset on spore morphology in *Platycerium*. Based on morphological characteristics, we classified the spores into four distinct types, each described in detail. Spore surface ornamentation proved to be an effective diagnostic feature for *Platycerium coronarium*, *P. madagascariense*, *P*. *ridleyi*, and *P*. *stemaria*. The systematic significance of spore morphology in *Platycerium* was discussed, while no significant linear correlation was found across *Platycerium* between spore sizes and genome sizes. Our findings are important for understanding the relationship between spores and ploidy levels in Polypodiaceae and their evolutionary implications.

## 1. Introduction

The genus *Platycerium* Desv. belongs to the family Polypodiaceae [1,2]. Morphologically, the leaves of *Platycerium* are strongly dimorphic, comprising a basal sterile leaf that clings to the substrate and a dichotomously forked trophosporophylls with coenosoroid to acrosoroid patches of sporangia [3,4,5]. *Platycerium* is known for its unique plant form and elegant posture [6]. With advantages such as strong stress tolerance and a long ornamental period, most species of the genus are often used as indoor hanging or wall-mounted ornamental plants [6,7]. Recently, utilizing genome-skimming sequencing, transcriptome sequencing, and flow cytometry, Zhao et al. [8] integrated evidence from plastomes, nuclear genes, ploidy levels, morphology, and geographic distribution to clarify the phylogeny and biogeographic history of *Platycerium*. Their study covered all 18 accepted native species and supported that *Platycerium* comprises three fully supported monophyletic clades: the Afro-American (AA) clade, which consists of seven species; the Javan-Australian (JA) clade with four species; and the Malayan-Asian (MA) clade comprising seven species. In addition, the AA and MA clades can be further divided into three and two subclades. In geographical distribution, the AA clade was only distributed in Africa, America, and Madagascar, but species of the JA clade and the MA clade were distributed in Southeast Asia and Oceania [8]. In addition, Zhao et al. [8] had detected that *Platycerium alcicorne* (P. Willemet) Desv. and *P. veitchii* (Underw.) C. Chr. might contain cryptic species, with *P. alcicorne* comprising *P. alcicorne*-Madagascar and *P. alcicorne*-Africa, and *P. veitchii* comprising *P. veitchii*-1 and *P. veitchii*-2. These cryptic species require further taxonomic evidence for verification.

Palynological data could provide important taxonomic evidence for plants [9,10]. In ferns, spore ornamentation varies among different lineages [9,11,12,13]. Species of the Pteridaceae typically have spherical spores with a trilete aperture and surface ornamentation that can be reticulate, ridged, or echinate, among others [11]. In contrast, spores of the Polypodiaceae possess a monolete aperture and exhibit relatively simple surface ornamentation, ranging from psilate to verrucate or tuberculate [9,12]. However, research on the spore morphology of *Platycerium* remains relatively limited. Tryon and Lugardon [5] were firstly observed *Platycerium* spores using scanning electron microscopy (SEM). They described six species: *Platycerium bifurcatum* (Cav.) C. Chr. and *P. stemaria* (P. Beauv.) Desv. had slightly papillate spore surfaces, but *P. andinum* Baker, *P. elephantotis* Schweinf., and *P. wallichii* Hooker exhibited short apertures and granules on their spore surfaces, while *P. ridleyi* Christ showed an irregular, somewhat echinate surface. Pérez-García et al. [14] described the spore morphological characteristics of *P. andinum* and *P. wandae* Raciborski. The average spore sizes of these two species were measured as 58 μm (length) × 35 μm (width), and the spore color of both species was weakly brown and lacked a perispore. However, the ornamentation of *P. andinum* exhibited a granulose surface, whereas *P. wandae* was smooth to slightly granulose. Jia [15] performed SEM on spores of *P. wallichii*, revealing that its spores were monolete and elliptical with granulate ornamentation. Nevertheless, a comprehensive and systematic study of *Platycerium* spores remains unavailable.

Within ferns, the relationship between spore sizes and ploidy levels has shown varied results. Passarelli et al. [16] and Li et al. [17] found a positive correlation between spore sizes and ploidy levels within *Blechnum* Linnaeus (64 samples) and *Gaga* Pryer, F.W. Li & Windham (44 samples). Sigel et al. [18], using 57 samples of *Argyrochosma* (J. Smith) Windham, revealed that spore size increased significantly with increasing ploidy. Recently, Gómez-Noguez et al. [19] measured spore size and mass across 23 fern species and found that among closely related fern species, the spore sizes of polyploids were usually significantly larger than those of diploids [20]. However, they also found no statistically significant correlation between spore sizes or mass and ploidy through statistical analysis. Another notable example was *Ceratopteris richardii* Brongn [21], which produces the largest fern spores (polar axis 89 μm, equatorial axis 126 μm, mass 800 ng). Its spore sizes were not associated with high ploidy [22] and were considered a species-specific adaptation strategy for aquatic environments [19]. Barrington et al. [23] found that in the genus *Dryopteris* Adanson, spores of diploid species [e.g., *D. fragrans* (Linnaeus) Schott, *D. dilatata* (Hoffmann) A. Gray] are inherently large, comparable in size to those of tetraploid species [e.g., *D. campyloptera* (Kunze) Clarkson]. This difference might be attributed to adaptations in diploids—such as dispersal or nutrient allocation—or environmental regulation, rather than ploidy. Similarly, the spore size of *Isoetes storkii* T.C. Palmer may be influenced by altitude and temperature, which could obscure the effect of ploidy [23]. These analyses indicated that spore size may be influenced by ecological adaptation and species-specific traits, and that ploidy might not be the sole determining factor [19,20,22,23]. Therefore, given the relative scarcity of chromosome count data for ferns [24] and the fact that both chromosome counting and flow cytometry require fresh plant materials, assessing ploidy via spore sizes retains significant biological relevance.

With these questions in mind, the objectives of this study are: (i) to clarify spore morphology of *Platycerium* using both Light Microscopy (LM) and SEM; (ii) to assess the phylogenetic signal of spore traits and their implications for species delimitation; and (iii) to evaluate the relationship between spore sizes and ploidy levels in *Platycerium*.

## 2. Materials and Methods

### 2.1. Taxon Sampling and Spore Observation

In addition to the 20 specimens of *Platycerium* previously used for both transcriptome and flow cytometry studies [8], we included 17 living materials cultivated in the greenhouses of Yunnan University and the Xishuangbanna Botanical Garden. In total, these 37 individuals represent all currently recognized 18 native species of *Platycerium*. Based on LM, we selected at least 20 well-developed spores from each material. For each material, spores were evenly mounted on sample stages using carbon tape, and the color was recorded. The stages were then placed in a BAL-TEC SCD005 vacuum ion sputter coated with a gold coating for 1.5 min. The coated samples were examined under a QUANTA 200 scanning electron microscope to examine spore morphology. Photographs were taken of the lateral, proximal, and distal surfaces and surface microstructures. The terminology used for spore morphological descriptions in this study follows established literature [5,9,12,13].

### 2.2. Genome Size Estimation and Phylogenetic Inference

Flow cytometry was employed to estimate the ploidy levels and genome sizes of *Platycerium* [8]. Experiments were conducted by the Molecular Biology Experiment Center (Germplasm Bank of Wild Species in Southwest China) using leaves of *Zea mays* L. B73 (2C = 2.96 pg) as the internal standard. The ploidy level and genome size of each sample were analyzed with a BD FACSCalibur flow cytometer. Phylogenetic reconstruction was performed using the Maximum Likelihood (ML) method based on a set of 812 single-copy nuclear genes identified by Zhao et al. [8] via transcriptome sequencing. The nucleotide substitution rate variation model was fitted using the ModelFinder tool under the corrected Akaike Information Criterion (AICc). ML analyses were conducted with 5000 ultrafast bootstraps replicates [25] in IQ-tree v2.1.3 [26].

### 2.3. Ancestral State Reconstruction and Correlation Analysis

To perform reconstruction of the ancestral state of morphology, we used the ML “fastAnc” method implemented with the “contMap” function in the R package “phytools v1.5-1” [27]. Three different models (equal-rates (ER), symmetric (SYM), and all-rates-different (ARD)) were fitted to the phylogenetic tree with the “fitDiscrete” command in the R package “geiger v2.0.11”, and the best models were selected by AICc. In total, three continuous characters (genome sizes, length of equatorial axis, and length of polar axis in spore) and two discrete traits of spore (colors and surfaces of ornamentation) were studied. In addition, we calculated the correlations between genome sizes and the equatorial and polar axis lengths of spores using the linear regression model “lm ()” function in R. Plots were generated using ggplot2 v2.2.1 [28].

## 3. Results

### 3.1. Spore Morphology of Platycerium

**Figure 1:**
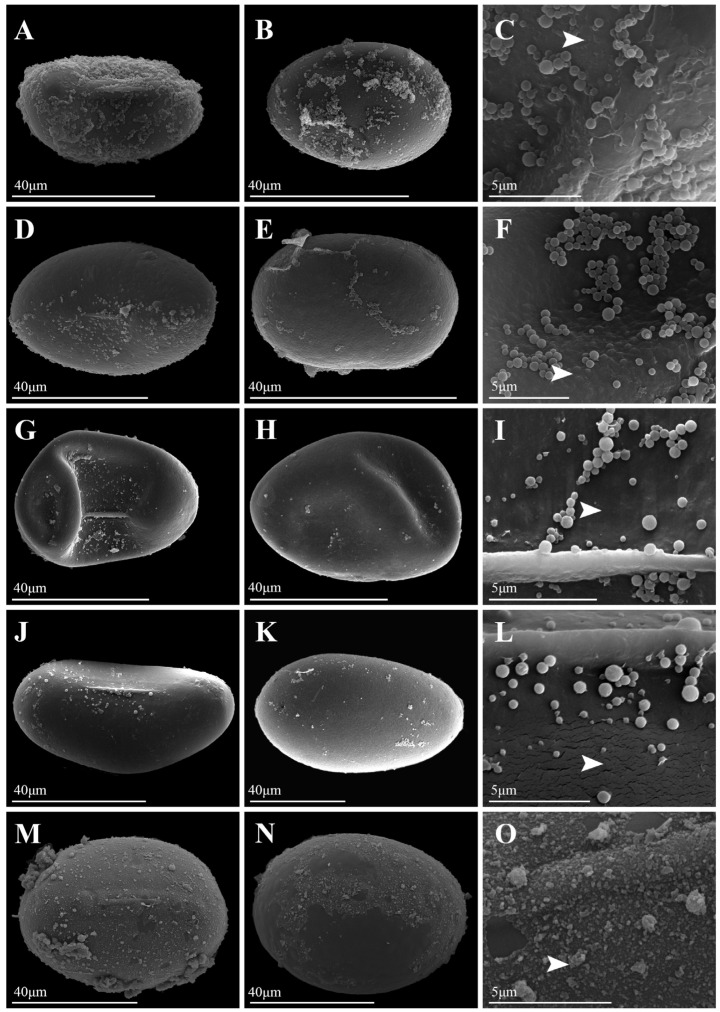
Spore morphology of *Platycerium*. (**A**–**C**) *P. alcicorne*-Africa. (**D**–**F**) *P. alcicorne*-Madagascar. (**G**–**I**) *P. andinum*. (**J**–**L**) *P. bifurcatum*. (**M**–**O**) *P. coronarium*. (**A**,**D**,**G**,**J**,**M**) Proximal surfaces; (**B**,**E**,**H**,**K**,**N**) Distal surfaces; (**C**,**F**,**I**,**L**,**O**) Portions of proximal surface enlarged to show infrastructural detail. White arrows indicate diagnostic ornamentation features.

**Figure 2:**
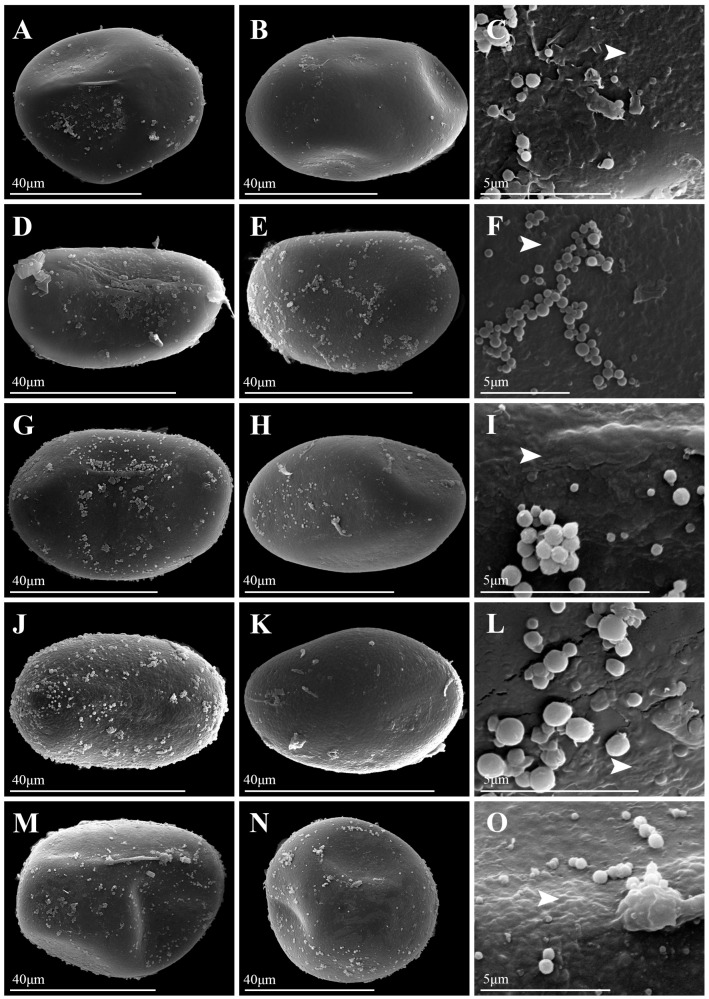
Spore morphology of *Platycerium*. (**A**–**C**) *P. elephantotis*. (**D**–**F**) *P. ellisii*. (**G**–**I**) *P. grande*. (**J**–**L**) *P. hillii*. (**M**–**O**) *P. holttumii*. (**A**,**D**,**G**,**J**,**M**) Proximal surfaces; (**B**,**E**,**H**,**K**,**N**) Distal surfaces; (**C**,**F**,**I**,**L**,**O**) Portions of proximal surface enlarged to show infrastructural detail. White arrows indicate diagnostic ornamentation features.

**Figure 3:**
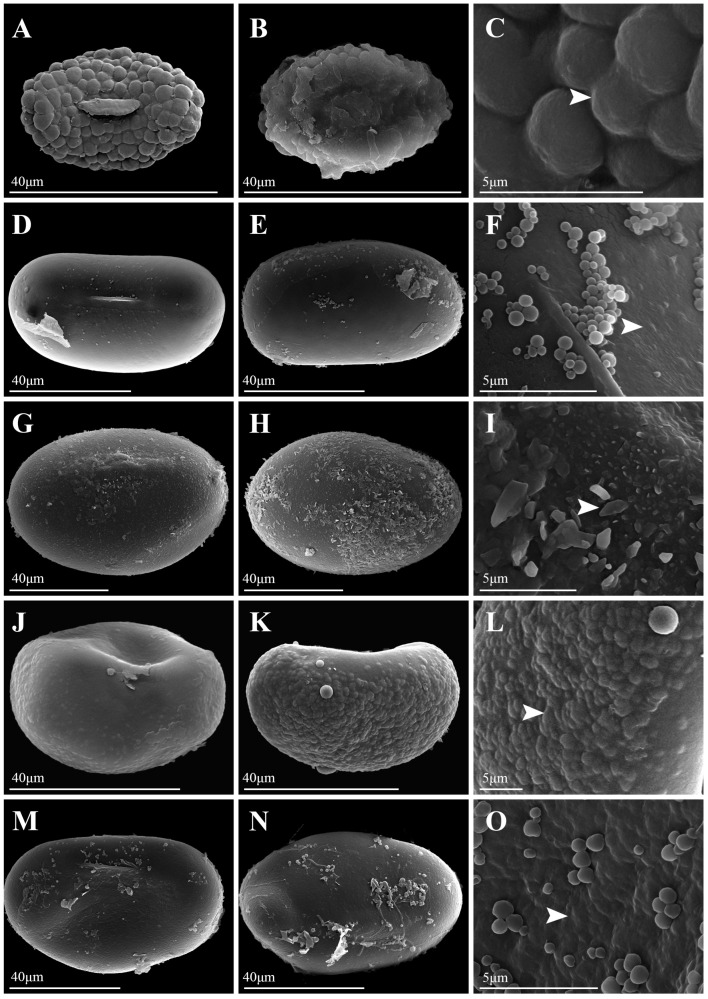
Spore morphology of *Platycerium*. (**A**–**C**) *P. madagascariense*. (**D**–**F**) *P. quadridichotomum*. (**G**–**I**) *P. ridleyi*. (**J**–**L**) *P. stemaria*. (**M**–**O**) *P. superbum*. (**A**,**D**,**G**,**J**,**M**) Proximal surfaces; (**B**,**E**,**H**,**K**,**N**) Distal surfaces; (**C**,**F**,**I**,**L**,**O**) Portions of proximal surface enlarged to show infrastructural detail. White arrows indicate diagnostic ornamentation features.

**Figure 4:**
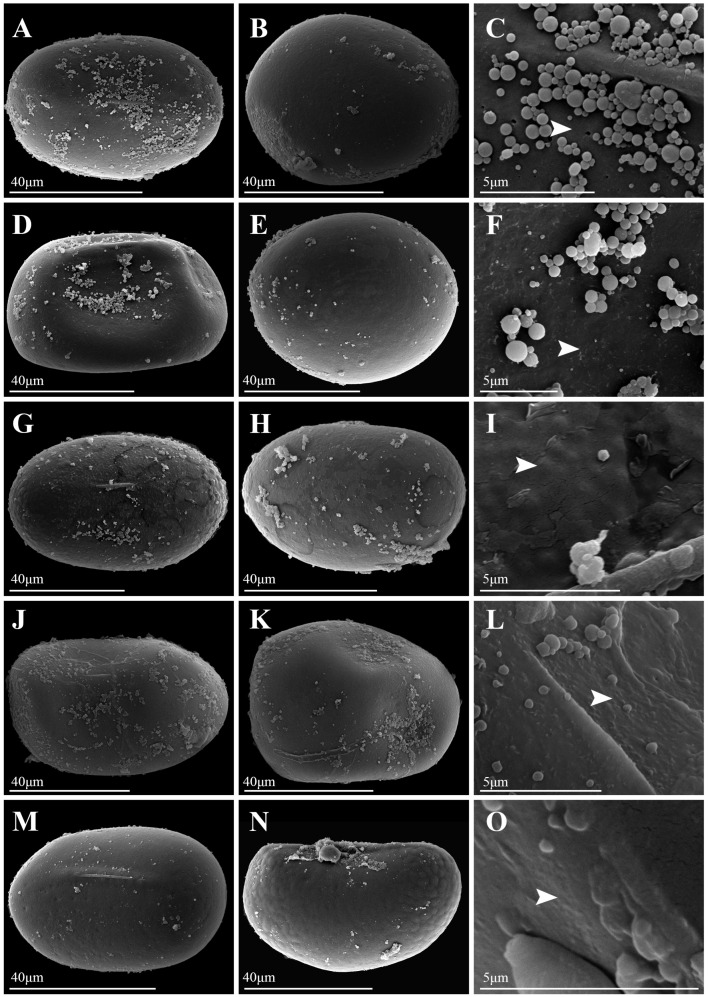
Spore morphology of *Platycerium*. (**A**–**C**) *P. veitchii*-1. (**D**–**F**) *P. veitchii*-2. (**G**–**I**) *P. wallichii*. (**J**–**L**) *P. wandae*. (**M**–**O**) *P. willinckii*. (**A**,**D**,**G**,**J**,**M**) Proximal surfaces; (**B**,**E**,**H**,**K**,**N**) Distal surfaces; (**C**,**F**,**I**,**L**,**O**) Portions of proximal surface enlarged to show infrastructural detail. White arrows indicate diagnostic ornamentation features.

Under LM, spores of *Platycerium* exhibited both plump and slightly flattened forms. LM images of all species were available in Figure S1. The spores of *P. andinum* were flattened, whereas the spores of the remaining species were well-developed and plump (Figure 1, Figure 2, Figure 3 and Figure 4). In addition, the spores of *Platycerium* were almost exclusively yellowish-brown in color, with occasional ones in yellowish-white (*P. ellisii* Baker) or brown (*P. superbum* de Jonch. & Hennipman, *P. wandae*) (Table 1).

SEM observations revealed that the spore wall ornamentations of *Platycerium* included verrucate, psilate, irregular granulate, and tuberculate types. A comparative schematic diagram of the four types of spore surface ornamentation is shown in Figure S2. Unlike the other three ornamentation types, the irregular granulate type lacked a perispore. The spores possessing a perispore were observed to have a thin perispore, which was abraded; granules were observed in all types, and we described the spores of 20 species in this genus (Table 1; Figure 1, Figure 2, Figure 3 and Figure 4). The spores were consistently bilateral and monolete (Figure 1, Figure 2, Figure 3 and Figure 4). The length of the spore aperture was typically one-third to one-half of the total spore length (Figure 1, Figure 2, Figure 3 and Figure 4). The equatorial view was elliptical or long-elliptical, while the polar view was reniform, bean-shaped, or elliptical (Figure 1, Figure 2, Figure 3 and Figure 4). Based on the structure and ornamentation of the spore wall, the spores of *Platycerium* can be divided into the following four types.

Type 1. Verrucate. The spores possess a perispore, with small verrucae and granules on the surface. Most species in this genus belong to this type, such as *Platycerium alcicorne* (Figure 1A–F), *P. elephantotis* (Figure 2A–C), *P. ellisii* (Figure 2D–F), and *P. grande* (A. Cunn. ex Hook.) J. Sm. (Figure 2G–I), *P. hillii* T. Moore (Figure 2J–L), *P. holttumii* de Jonch. & Hennipman (Figure 2M–O), *P. quadridichotomum* (Bonap.) Tardieu (Figure 3D–F), *P. superbum* (Figure 3M–O), *P. wallichii* (Figure 4G–I), *P. wandae* (Figure 4J–L), *P. willinckii* (T. Moore) Hennipman & M.C. Roos (Figure 4M–O).

Type 2. Psilate. The spores lack a perispore and have a psilate surface with granules, such as *Platycerium andinum* (Figure 1G–I), *P. bifurcatum* (Figure 1J–L), *P. veitchii* (Figure 4A–F).

Type 3. Irregular Granulate. The spores had a perispore, densely covered with irregular granular protrusions on the surface, as seen in *Platycerium coronarium* (D. Koenig ex O.F. Müll.) Desv. (Figure 1M–O). In addition to granules, the spore surfaces also bore lamellate, clastic structures, as observed in *P. ridleyi* (Figure 3G–I).

Type 4. Tuberculate. The spores possess a perispore that is covered with regular tubercles. This characteristic was distinct in *Platycerium madagascariense* Baker (Figure 3A–C), whose spore surface was uniformly covered with large, flat tuberculate protrusions. The spores of *P. stemaria* also had small tubercles and granules on the surface (Figure 3J–L).

### 3.2. Spore Sizes of Platycerium

**Figure 5:**
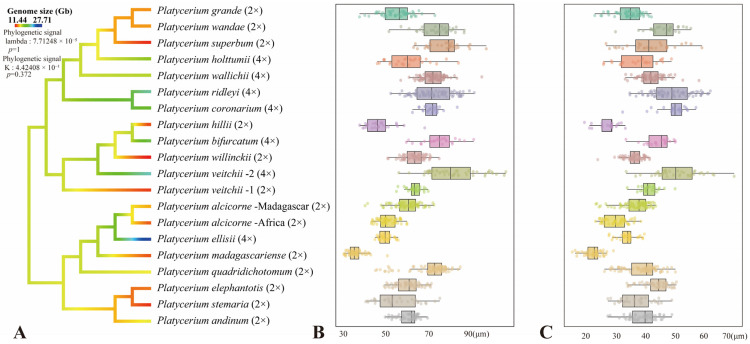
Ancestral state reconstruction of genome sizes and boxplots of spore morphological characteristics of *Platycerium*. (**A**) Genome size. (**B**) Equatorial axis. (**C**) Polar axis.

**Figure 6:**
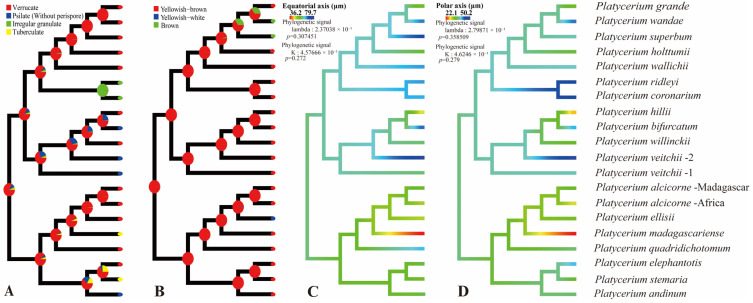
Ancestral spore states reconstruction of *Platycerium*. (**A**) Spore type. (**B**) Spore color. (**C**) Equatorial axis length. (**D**) Polar axis length.

The original measurement data for all species were available in Table S1. The smallest spores measured in this study were *Platycerium madagascariense*, with an equatorial axis length of 31.0–50.3 μm and a polar axis length of 15.1–28.9 μm (Table 1). The largest spores were from *P. veitchii*-2, with an equatorial axis length of 55.4–103.0 μm and a polar axis length of 33.2–69.5 μm (Table 1). The average spore equatorial axis length of *Platycerium* ranges from 50 μm to 70 μm, while the average polar axis length ranges from 30 μm to 50 μm (Table 1; Figure 5B,C). Within the AA clade, the largest spores belong to *P. quadridichotomum*, measuring 69.2 × 38.7 μm, and the smallest spores are from *P. madagascariense*, measuring 36.2 × 22.1 μm (Table 1; Figure 5B,C). In the JA clade, the largest spores are those of *P. veitchii*-2, measuring 79.7 × 50.2 μm, while the smallest spores are from *P. hillii*, measuring 47.0 × 27.0 μm (Table 1; Figure 5B,C). Within the MA clade, the largest spores belong to *P. superbum*, measuring 76.0 × 41.1 μm, and the smallest spores are from *P. grande*, measuring 54.6 × 34.0 μm (Table 1; Figure 5B,C). Additionally, the equatorial and polar axes of spores in the AA clade are smaller than those in the JA and MA clades (Figure 5B,C and Figure 6C,D).

### 3.3. Phylogeny and Genome Sizes

A total of 812 single-copy nuclear genes shared by all 20 individuals were used to infer the phylogeny of *Platycerium* (Figure 5A). The genome sizes range from 11.44 Gb in *Platycerium willinckii* to 27.71 Gb in *P. ellisii* (Table 1; Figure 5A). The genome sizes of diploid species in *Platycerium* range from 11.44 Gb (*P. willinckii*) to 15.42 Gb (*P. andinum*), while tetraploid species have genome sizes approximately ranging from 16.35 Gb (*P. wallichii*) to 27.71 Gb (*P. ellisii*) (Table 1; Figure 5A). Both diploid and tetraploid species were found concurrently within the AA clade, JA clade, and MA clade. The majority of species were diploid (*P. alcicorne*, *P. andinum*, *P. elephantotis*, *P. grande*, *P. hillii*, *P. madagascariense*, *P. quadridichotomum*, *P. stemaria*, *P. superbum*, *P. veitchii*-1, *P. wandae*, and *P. willinckii*). In addition, there were seven tetraploid species (*P. bifurcatum*, *P. coronarium*, *P. ellisii*, *P. holttumii*, *P. ridleyi*, *P. veitchii*-2, and *P. wallichii*). Furthermore, different individuals of *P. veitchii* include both diploids (*P. veitchii*-1, genome size = 12.24 Gb) and tetraploids (*P. veitchii*-2, genome size = 22.56 Gb). Ancestral genome size reconstruction indicated that the ancestral genome size of *Platycerium* was approximately 12 Gb, with a weak phylogenetic signal (lambda = 7.71248 × 10^−5^, *p* = 1; K = 4.42408 × 10^−1^, *p* = 0.372). Diploidy was inferred as the ancestral ploidy state (Figure 5A).

### 3.4. Ancestral Character State Reconstruction

Four spore characteristics of *Platycerium* (spore type, spore color, equatorial axis length, and polar axis length) were selected for ancestral state reconstruction (Figure 6). The reconstruction of ancestral spore type indicated that verrucate ornamentation is the ancestral state for spores in this genus, with all other ornamentation types being derived (Figure 6A). In addition, the AA clade exhibits the greatest diversity of spore ornamentation types, including verrucate, psilate, and tuberculate (Figure 6A). The reconstruction of ancestral spore color revealed that yellowish-brown was the ancestral state for spores in *Platycerium*, while yellowish-white and brown were identified as derived states (Figure 6B). The reconstruction of the ancestral equatorial and polar axis sizes of *Platycerium* indicated that the equatorial axis size (lambda = 2.37038 × 10^−1^, *p* = 0.307451; K = 4.57666 × 10^−1^, *p* = 0.272; Figure 6C) was approximately 60 μm, and the polar axis length (lambda = 2.79871 × 10^−1^, *p* = 0.358509; K = 4.6246 × 10^−1^, *p* = 0.279; Figure 6D) was approximately 40 μm.

### 3.5. The Relationships Between Genome Sizes and Spore Sizes

**Figure 7:**
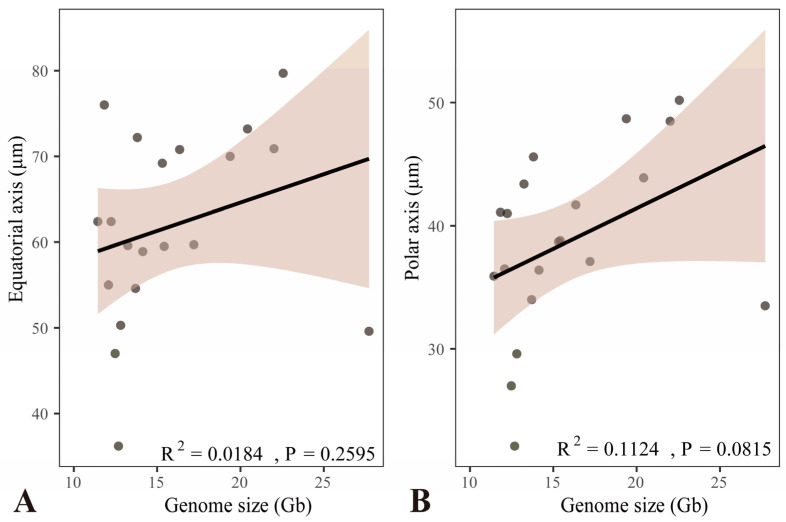
Linear regression analysis of spore sizes and genome sizes in *Platycerium*. (**A**) Equatorial axis. (**B**) Polar axis.

Linear regression analysis revealed no significant relationship between genome size and either the equatorial or polar spore axis length in *Platycerium* (Figure 7). The analysis of the equatorial axis versus genome size showed R^2^ = 0.0184 and *p* = 0.2595 (Figure 7A), while the polar axis versus genome size showed R^2^ = 0.1124 and *p* = 0.0815 (Figure 7B). Both R^2^ were well below 0.5, indicating that genome size has very weak explanatory power for spore axis length, and the strength of the linear association is very low (Figure 7A,B). The *p*-values did not reach the significance level, which indicates that the linear association between genome size and spore axis length is not statistically significant (Figure 7A,B). In conclusion, the analysis demonstrated that spore size and genome size in *Platycerium* are not significantly linearly correlated (Figure 7).

## 4. Discussion

### 4.1. General Features and Stability of Platycerium Spore

This study documented substantial intraspecific variation in spore morphology among *Platycerium*, particularly in spore size and surface ornamentation (Table 1; Figure 1, Figure 2, Figure 3 and Figure 4). The spores of all species are elliptic or oblong in equatorial view and bean-shaped, kidney-shaped, or elliptic in polar view. They are monolete and possess a thin, abraded perispore. The surface ornamentation is formed by either the perispore or the exospore. These observations are consistent with previous studies on spore morphology in Polypodiaceae [9,12].

Colors and sizes: —Based on observations under LM, the spore colors of each species are observed and described in Table 1, including yellowish-brown, yellowish-white, and brown. Spore color was relatively stable and therefore had limited value for the taxonomy of *Platycerium*. Pérez-García et al. [14] reported weakly brown spores in *Platycerium andinum* and *P. wandae*, with average spore sizes of 58 μm × 35 μm. In this study, *P. andinum* spores were observed to be yellowish-brown, with an average size of 59.5 μm × 38.8 μm (Table 1), consistent with Pérez-García et al. [14]. However, the spores of *P. wandae* were brown, with an average size of 72.2 μm × 45.6 μm (Table 1). Our results indicate that yellowish-brown spore color is stable within the genus, and we documented spore size for each specimen (Table 1). We found that spore size varied continuously and was less stable. Additionally, ancestral state reconstruction revealed that yellowish-brown was the ancestral spore color in *Platycerium*, while yellowish-white and brown colors evolved independently twice (Figure 6A). The ancestral spore size was approximately 60 μm along the equatorial axis and 40 μm along the polar axis, followed by multiple independent evolution changes.

Surface ornamentations: —In contrast to the slightly papillate spore surfaces reported for *P. bifurcatum* and *P. stemaria* by Tryon and Lugardon [5], our study finds that *P. bifurcatum* has psilate ornamentation without perispore (Figure 1J–L), while *P. stemaria* tuberculate ornamentation with perispore (Figure 3J–L). Tryon and Lugardon [5] introduced that *P. andinum*, *P. elephantotis*, and *P. wallichii* exhibited short apertures and granules on their spore surfaces. Pérez-García et al. [14] reported that *P. andinum* and *P. wandae* lack a perispore and have granular material on the spore surface. Jia [15] performed SEM on spores of *P. wallichii* and revealed monolete, elliptical spores with granulate ornamentation. We agree with their description of granules on the spore surface for these species but provide a revised description: *P. andinum* lacks a perispore and has a psilate surface (Figure 1G–I), whereas *P. elephantotis* (Figure 2A–C), *P. wallichii* (Figure 4G–I), and *P. wandae* (Figure 4J–L) have a perispore with a verrucate surface. A previous study described *P. ridleyi* as having an irregular, somewhat echinate spore surface [5]. We also consider this a distinctive type of ornamentation and therefore refine its description as follows: *P. ridleyi* has a perispore with an irregular granulate surface featuring lamellate, clastic structures (Figure 3G–I). Based on detailed observations of spore ornamentation using SEM, the spores of *Platycerium* can be classified into four types (as mentioned above).

Based on the three core clades (AA, JA, and MA) defined by Zhao et al. [8], spore ornamentation does not exhibit particularly distinct differences across these major lineages. Within the AA clade, spore ornamentation includes verrucate, psilate, and tuberculate types. The tuberculate ornamentation is unique to this clade, particularly evident in *P. madagascariense* (Figure 3A–C), which possesses large, flat tubercles. In contrast, *P. stemaria* (Figure 3J–L) exhibits small tubercles with granules on the surface. In the AA clade, only the spores of *P. andinum* (Figure 1G–I) lack a perispore but have a psilate surface with granules, whereas the remaining species have small verrucae and granules (Figure 1A–F, Figure 2A–F and Figure 3D–F). In the JA clade, spore ornamentation comprises verrucate and psilate types. The spores of *P. hillii* (Figure 2J–L) and *P. willinckii* (Figure 4M–O) have small verrucae and granules, whereas those of *P. bifurcatum* (Figure 1J–L), *P. veitchii*-1 (Figure 4A–C), and *P. veitchii*-2 (Figure 4D–F) lack a perispore but have a psilate surface with granules. The MA clade exhibits verrucate and irregular granulate ornamentation. The spore surface ornamentation of *P. coronarium* (Figure 1M–O) and *P. ridleyi* (Figure 3G–I) is particularly distinctive and easily recognizable. The granular ornamentation is modified, characterized by irregular granules with clastic structures. Other species in this clade exhibit small verrucae and granules (Figure 2G–I,M–O, Figure 3M–O and Figure 4G–L). In summary, spore ornamentation observed under SEM clearly distinguishes *P. coronarium* (Figure 1M–O), *P. madagascariense* (Figure 3A–C), *P. ridleyi* (Figure 3G–I), and *P. stemaria* (Figure 3J–L). Our observations indicate that the most prevalent ornamentation in *Platycerium* spores is verrucate. Granules are present across all clades, with variations occurring at specific levels.

### 4.2. Systematic Significance of Spore Morphology of Platycerium

In ferns, spores of closely related polyploids are generally significantly larger than those of diploids [16,18,19,20,23]. This study found that spores of tetraploids were larger than those of diploids only within the JA clade (Figure 5). The spores of *P. bifurcatum* (tetraploid) were larger than those of its closest relative, *P. hillii* (diploid) (Figure 5A–C). The spores of *P. veitchii*-2 (tetraploid) were larger than those of the closely related diploids *P. hillii* and *P. willinckii*. However, in the AA clade and the MA clade, the spores of the remaining tetraploids (*P. coronarium*, *P. ellisii*, *P. holttumii*, *P. ridleyi*, and *P. wallichii*) were smaller than those of their closely related diploid individuals (Figure 5). Zhao et al. [8] suggested that *P. alcicorne* and *P. veitchii* may contain cryptic species. Our observations confirmed the existence of two ploidy variants in *P. veitchii*: *P. veitchii*-1 (diploid) and *P. veitchii*-2 (tetraploid) (Table 1; Figure 5A). Although they shared the same type of spore surface ornamentation (Figure 4A–F), their spore sizes differed significantly. The spores of *P. veitchii*-1 measured 62.4 × 41.0 µm, while those of *P. veitchii*-2 measured 79.7 × 50.2 µm (Table 1; Figure 5B,C and Figure 6C,D). In contrast, all observed individuals of *P. alcicorne* were diploid (Table 1; Figure 5A), exhibited uniform spore ornamentation (Figure 1A–F), and showed almost no variation in spore sizes (Table 1; Figure 5B,C and Figure 6C,D). Therefore, *P. alcicorne* and *P. veitchii* contain cryptic lineages which were supported by both phylogeny and cytology, and *P. veitchii*-1 and *P. veitchii*-2 can be distinguished based on spore size observation. However, the spore morphology alone cannot reliably differentiate *P. alcicorne*-Africa from *P. alcicorne*-Madagascar; effective identification requires integrating more evidence from cytology, molecular, and other data.

Ancestral state reconstruction of spore surface ornamentation provided evidence for the classification of *Platycerium*. Zhao et al. [8] confirmed that the AA clade and the MA clade could be further divided into three and two subclades. Within the AA clade, the subclade comprising *P. elephantotis* (Figure 2A–C and Figure 6A), *P. stemaria* (Figure 3J–L and Figure 6A), and *P. andinum* (Figure 1G–I and Figure 6A) was distinguished by their spore surface ornamentation. In the MA clade, distinct spore ornamentations characterized the two subclades: species in the first subclade all exhibited verrucate spore ornamentation, including *P. grande*, *P. holttumii*, *P. superbum*, *P. wallichii*, and *P. wande*, while those in the second subclade, composed of *P. ridleyi* and *P. coronarium,* all had irregular granulate ornamentation (Figure 6A). Spore color is generally considered to have limited taxonomic significance [29] and can not serve as an interspecific diagnostic character in *Platycerium* (Figure 6B). The AA clade possessed the smallest spores, with both equatorial and polar axis measurements generally smaller than those in the other two clades (Figure 5B,C and Figure 6C,D), consistent with the phylogenetic analysis by Zhao et al. [8].

Barrington et al. [20] demonstrated a strong positive correlation between spore size and genome size in *Polystichum* Roth and *Adiantum* L., but only under the condition that ploidy directly determines genome size within closely related species or species complexes. In our study, we observed that all tetraploid spores are larger than diploid ones within the JA clade, which implied a potential positive correlation between spore size and genome size in this lineage (Table 1). However, across the entire genus of *Platycerium*, no significant linear correlation was found between genome size and spore size (Figure 7). The tetraploid *Platycerium ellisii* had the largest genome (27.71 Gb) (Table 1; Figure 5) but did not have the largest spores, which measured 49.6 × 33.5 µm. The largest spores were found in *P. veitchii*-2 (79.7 × 50.2 µm), which had a genome size of 22.56 Gb (Table 1; Figure 5). Therefore, we propose that spore size is likely influenced by multiple factors, such as altitude, temperature, and species-specific characteristics. Ploidy and genome size may not be the decisive factors determining spore size [19,20,22,23].

## 5. Conclusions

This study established the first comprehensive spore morphological dataset for the genus *Platycerium*, including analyses of the proximal surfaces, distal surfaces, and surface ornamentation of the spores (Figure 1, Figure 2, Figure 3 and Figure 4). Statistical and phylogenetic analyses were conducted on spore color, types, and genome sizes within *Platycerium* (Figure 5, Figure 6 and Figure 7). These characteristics proved useful for species distinction and classification. For example, distinct spore ornamentation can distinguish *P. coronarium* (Table 1; Figure 1M–O), *P. madagascariense* (Table 1; Figure 3A–C), *P. ridleyi* (Table 1; Figure 3G–I), and *P. stemaria* (Table 1; Figure 3J–L). Additionally, spore equatorial and polar dimensions clearly showed that the AA clade has the smallest spores, with values generally lower than those of the other two clades (Figure 5B,C and Figure 6C,D). In addition, within the JA clade, polyploid species closely related to diploids typically exhibit significantly larger spores (Figure 5). However, we observed that spore color has limited taxonomic value in this genus, and no significant linear correlation was found between spore size and genome size (Figure 7).

## Figures and Tables

**Table 1:** Characteristics of spore morphology and genome size of *Platycerium* included in this study.

Species	Spore Color	Ploidy Level	Genome Size (Gb)	Spore Equatorial × Polar Axis (µm)	Ornamentation	Figure
*Platycerium alcicorne*-Africa	Yellowish-brown	Diploid	12.81	(43.2–59.0) 50.3 × 29.6 (22.9–38.2)	With perispore, small verrucae, and abundant granules	Figure 1A–C
*P. alcicorne*-Madagascar	Yellowish-brown	Diploid	14.14	(40.4–71.1) 58.9 × 36.4 (23.0–43.2)	With perispore, small verrucae, and granules	Figure 1D–F
*P. andinum*	Yellowish-brown	Diploid	15.42	(49.4–68.3) 59.5 × 38.8 (27.0–48.6)	Without a perispore, with a psilate surface and granules	Figure 1G–I
*P. bifurcatum*	Yellowish-brown	Tetraploid	20.42	(58.8–88.8) 73.2 × 43.9 (33.1–49.7)	Without a perispore, with a psilate surface and granules	Figure 1J–L
*P. coronarium*	Yellowish-brown	Tetraploid	19.38	(61.5–81.5) 70.0 × 48.7 (32.0–56.6)	With perispore, irregular granulate with clustered, clastic structures	Figure 1M–O
*P. elephantotis*	Yellowish-brown	Diploid	13.24	(48.9–70.0) 59.6 × 43.4 (33.4–50.1)	With perispore, small verrucae, and granules	Figure 2A–C
*P. ellisii*	Yellowish-white	Tetraploid	27.71	(44.8–55.2) 49.6 × 33.5 (27.4–39.3)	With perispore, small verrucae, and granules	Figure 2D–F
*P. grande*	Yellowish-brown	Diploid	13.70	(38.0–71.5) 54.6 × 34.0 (22.6–41.7)	With perispore, small verrucae, and granules	Figure 2G–I
*P. hillii*	Yellowish-brown	Diploid	12.48	(38.0–67.0) 47.0 × 27.0 (19.2–32.9)	With perispore, small verrucae, and granules	Figure 2J–L
*P. holttumii*	Yellowish-brown	Tetraploid	17.20	(45.9–82.0) 59.7 × 37.1 (25.6–48.4)	With perispore, small verrucae, and granules	Figure 2M–O
*P. madagascariense*	Yellowish-brown	Diploid	12.68	(31.0–50.3) 36.2 × 22.1 (15.1–28.9)	With perispore, large and flat tubercles	Figure 3A–C
*P. quadridichotomum*	Yellowish-brown	Diploid	15.31	(45.1–82.8) 69.2 × 38.7 (25.9–49.8)	With perispore, small verrucae, and granules	Figure 3D–F
*P. ridleyi*	Yellowish-brown	Tetraploid	22.01	(51.9–89.0) 70.9 × 48.5 (34.4–61.5)	With perispore, irregular granulate with lamellate, clastic structures	Figure 3G–I
*P. stemaria*	Yellowish-brown	Diploid	12.08	(40.7–73.7) 55.0 × 36.5 (27.0–48.6)	With perispore, small tubercles, and granules	Figure 3J–L
*P. superbum*	Brown	Diploid	11.83	(62.0–94.4) 76.0 × 41.1 (26.4–58.6)	With perispore, small verrucae, and granules	Figure 3M–O
*P. veitchii*-1	Yellowish-brown	Diploid	12.24	(54.4–68.5) 62.4 × 41.0 (33.5–49.6)	Without a perispore, with a psilate surface and granules	Figure 4A–C
*P. veitchii*-2	Yellowish-brown	Tetraploid	22.56	(55.4–103.0) 79.7 × 50.2 (33.2–69.5)	Without a perispore, with a psilate surface and granules	Figure 4D–F
*P. wallichii*	Yellowish-brown	Tetraploid	16.35	(56.3–92.4) 70.8 × 41.7 (32.9–57.7)	With perispore, small verrucae, and granules	Figure 4G–I
*P. wandae*	Brown	Diploid	13.81	(51.1–84.6) 72.2 × 45.6 (29.6–54.9)	With perispore, small verrucae, and granules	Figure 4J–L
*P. willinckii*	Yellowish-brown	Diploid	11.44	(50.5–73.4) 62.4 × 35.9 (23.6–41.1)	With perispore, small verrucae, and granules	Figure 4M–O

## Data Availability

The original contributions presented in this study are included in the article/Supplementary Material. Further inquiries can be directed to the corresponding authors.

## Supplementary Materials

The following supporting information can be downloaded at: https://www.mdpi.com/article/10.3390/plants15030370/s1, Table S1: Raw measurement data of spore sizes of *Platycerium*. Figure S1: Spore morphology of *Platycerium* species under LM. All scale bars represent 0.1 mm. The black arrows indicate the target spores. (A) *Platycerium alcicorne*-Africa, (B) *P. alcicorne*-Madagascar, (C) *P. andinum*, (D) *P. bifurcatum*, (E) *P. coronarium*, (F) *P. elephantotis*, (G) *P. ellisii*, (H) *P. grande*, (I) *P. hillii*, (J) *P. holttumii*, (K) *P. madagascariense*, (L) *P. quadridichotomum*, (M) *P. ridleyi*, (N) *P. stemaria*, (O) *P. superbum*, (P) *P. veitchii*-1, (Q) *P. veitchii*-2, (R) *P. wallichii*, (S) *P. wandae*, (T) *P. willinckii*. Figure S2: The four distinct types of spore surface ornamentation observed under SEM in *Platycerium*, with white arrows indicating key diagnostic characteristics. (A) Verrucate: *Platycerium alcicorne*-Madagascar. (B) Psilate: *P. bifurcatum*. (C) Irregular granulate: *P. coronarium*. (D) Tuberculate: *P. madagascariense*.
