# Supplementary figures and images for: Spore Morphology of Platycerium (Polypodiaceae) and Its Implications
Source: Plants (Basel). 2026 Jan 24;15(3):370. doi: 10.3390/plants15030370 (PMC12899769; doi:10.3390/plants15030370)

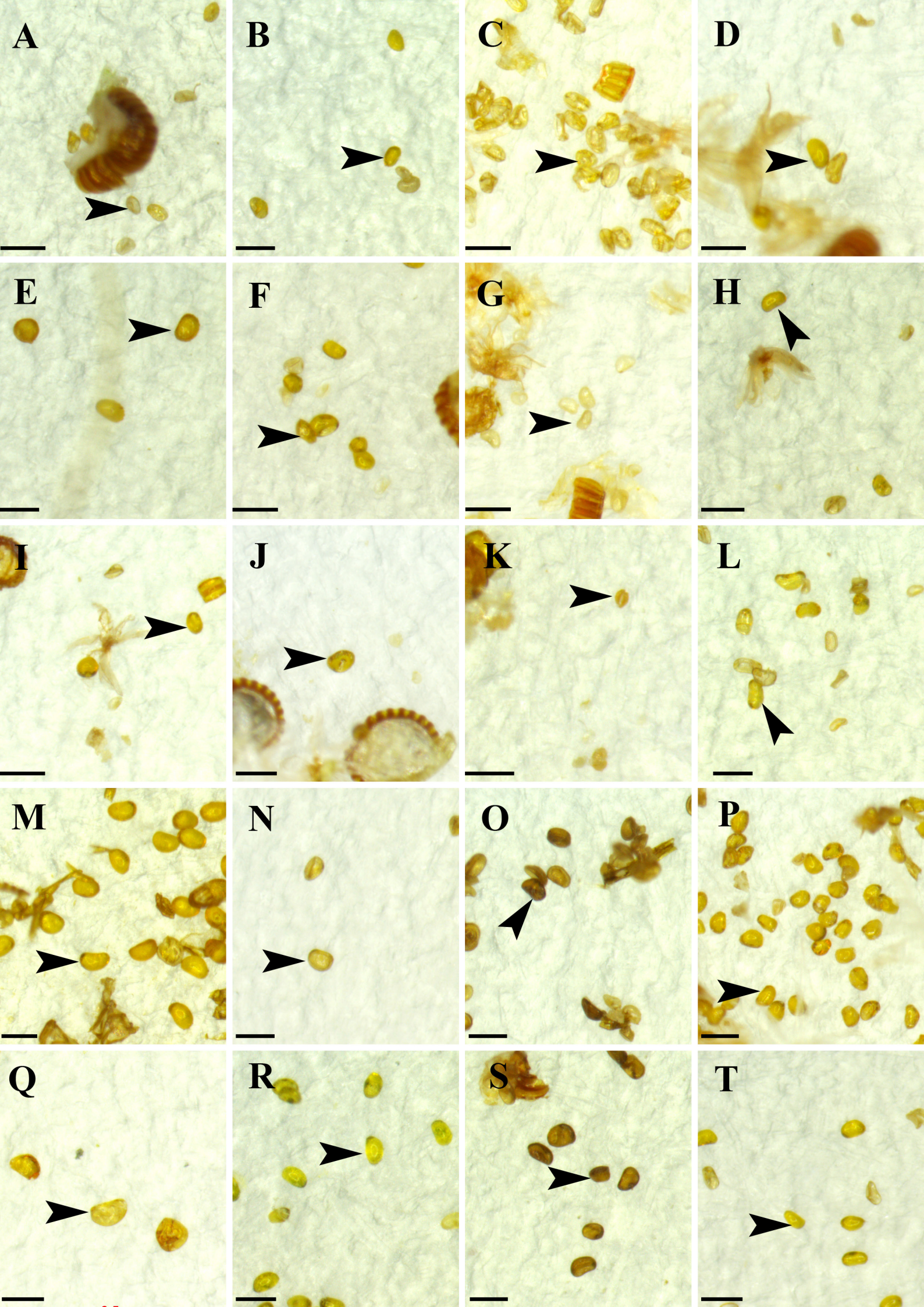

Supplement: Supplementary file 1 [file plants-15-00370-s001.zip › Supplementary File/FigureS1.pdf]

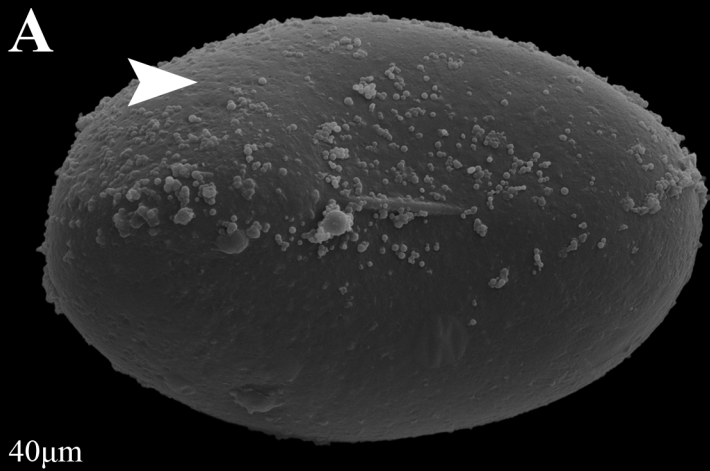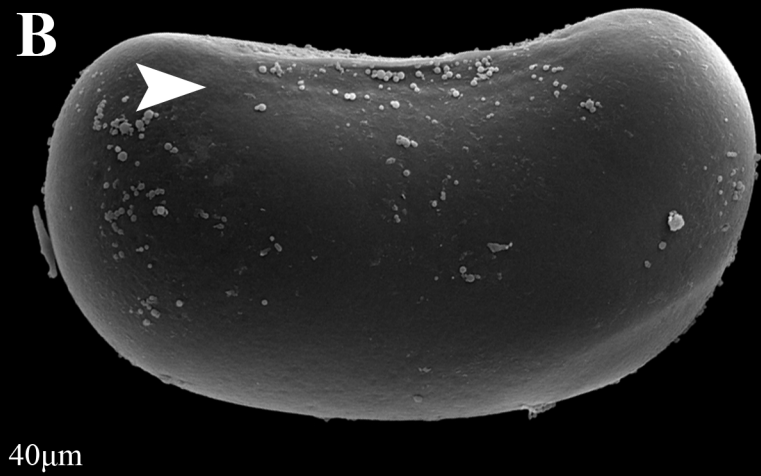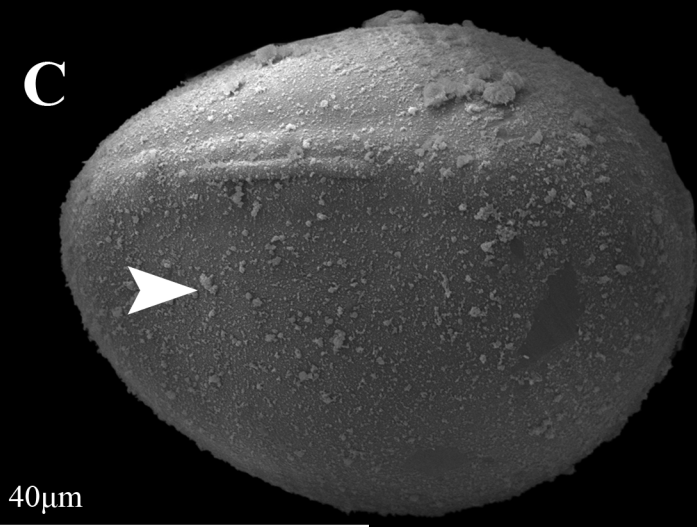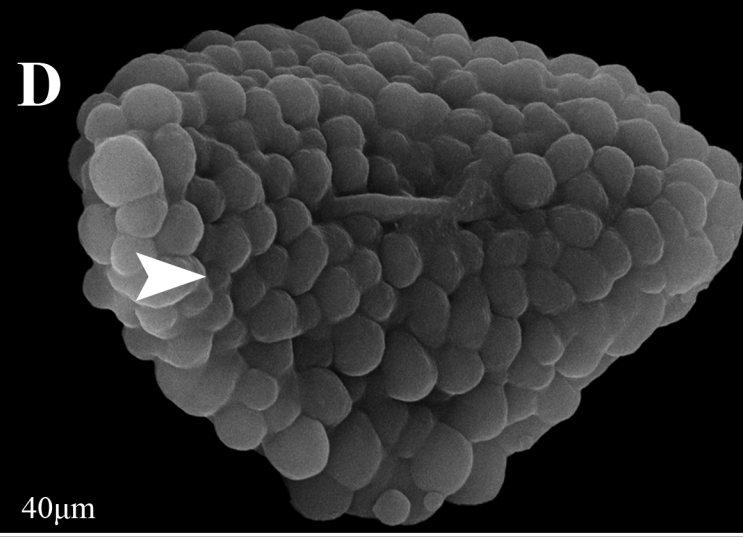

Supplement: Supplementary file 1 [file plants-15-00370-s001.zip › Supplementary File/FigureS2.pdf]
